# Differential effects of dietary flavonoids on adipogenesis

**DOI:** 10.1007/s00394-018-1663-8

**Published:** 2018-03-14

**Authors:** Manizheh Khalilpourfarshbafi, Khadijeh Gholami, Dharmani Devi Murugan, Munavvar Zubaid Abdul Sattar, Nor Azizan Abdullah

**Affiliations:** 1 Department of Pharmacology, Faculty of Medicine, University of Malaya, 50603 Kuala Lumpur, Malaysia; 2 Division of Human Biology, School of Medicine, International Medical University, 57000 Kuala Lumpur, Malaysia; 3 Faculty of Pharmacy, MAHSA University, 42610 Jenjarum, Malaysia

**Keywords:** Adipogenesis, Flavonoids, Obesity, Hyperplasia, Adipocyte

## Abstract

**Propose:**

Obesity is a fast growing epidemic worldwide. During obesity, the increase in adipose tissue mass arise from two different mechanisms, namely, hyperplasia and hypertrophy. Hyperplasia which is the increase in adipocyte number is characteristic of severe obese patients. Recently, there has been much interest in targeting adipogenesis as therapeutic strategy against obesity. Flavonoids have been shown to regulate several pathways and affect a number of molecular targets during specific stages of adipocyte development.

**Methods:**

Presently, we provide a review of key studies evaluating the effects of dietary flavonoids in different stages of adipocyte development with a particular emphasis on the investigations that explore the underlying mechanisms of action of these compounds in human or animal cell lines as well as animal models.

**Results:**

Flavonoids have been shown to regulate several pathways and affect a number of molecular targets during specific stages of adipocyte development. Although most of the studies reveal anti-adipogenic effect of flavonoids, some flavonoids demonstrated proadipogenic effect in mesenchymal stem cells or preadipocytes.

**Conclusion:**

The anti-adipogenic effect of flavonoids is mainly via their effect on regulation of several pathways such as induction of apoptosis, suppression of key adipogenic transcription factors, activation of AMPK and Wnt pathways, inhibition of clonal expansion, and cell-cycle arrest.

## Introduction

Obesity, which can be defined as increased body mass index (greater than 30 kg/m^2^), has been identified as a risk factor for the pathogenesis of many chronic diseases including cancer, hypertension, osteoarthritis, and cardiovascular diseases. It is also closely linked with metabolic disorders including insulin resistance and type 2 diabetes mellitus (T2DM) [1]. Obesity has been considered the fastest growing epidemic worldwide. According to the World Health Organization, in year 2014, more than 1.9 billion adults were overweight of which over 600 million were obese [2]. In the United States, the prevalence of adult obesity is greater than one-third (34.9%) of the population [3].

In obesity, the increase in adipose tissue mass arise via two main distinct mechanisms, increasing adipocyte number (hyperplasia) and/or increasing adipocyte volume (hypertrophy) [4, 5]. Hypertrophy occurs in overweight individuals and prolonged period of weight gain in adulthood leads to hyperplasia. Hyperplasia is mostly associated with severity of obesity and is the characteristic of morbidly obese individuals [6]. Hyperplasia takes place through adipogenesis that involves a cascade of transcriptional factors and cell-cycle proteins which leads to development of mature adipocyte [7]. This process can be divided into three main phases: growth arrest, clonal expansion, and terminal differentiation. Inhibition of adipocyte differentiation by interrupting any of these stages may serve as potential therapeutic strategy against adipogenesis and hence obesity.

Pharmaceutical approaches for weight management include altering metabolism, appetite, or fat absorption. Currently available drugs such as central nervous system stimulants, or peripherally acting anti-obesity drugs, are associated with several adverse effects such as hyperthyroidism, palpitations, anxiety, insomnia, and diarrhea [8]. The development of new and safe anti-obesity agent has become a necessity. Several studies have shown the potential of natural products to counteract obesity. Flavonoids represent the most researched groups of phytochemicals with regards to their effects on weight management. Studies have shown fruits and vegetables rich in several flavonoid subclasses, particularly flavonols, anthocyanins, and flavones are associated with less weight gain. A study which assessed the associations between habitual consumption of all flavonoid subclasses and weight gain among 124,086 American men and women over a period of 24 years showed higher intake of foods rich in flavonols, flavan-3-ols, anthocyanins, and flavonoid polymers may contribute to weight maintenance in adulthood after adjustment for changes in other lifestyle factors such as diet, smoking status, and physical activity [9].Several other studies on human and rodents provide evidence that flavonoids can cause suppression of appetite[10–12], increase glucose uptake in muscle [13], decrease fat absorption [14], and inhibit adipogenesis [15, 16].

A prospective cohort study indicates that obesity is associated with shorten life expectancy and indeed this study divulges that obesity in adulthood is a powerful predictor of death at older ages [17]. Flavonoids have been reported to affect health and life span of various model organisms through different mechanisms including energy-restriction like effects [18]. In this context, some of the molecular targets of the anti-adipogenic effects of flavonoids which overlap with some energy-restriction mimetics could be in part explain their lifespan extending properties [18].

This review summarizes the mechanisms of adipogenesis and highlights the anti-adipogenic effect of flavonoids and their corresponding underlying mechanisms of actions.

### Overview of adipogenesis

Adipogenesis occurs in two differentiation stages in which an undifferentiated multipotent mesenchymal stem cell transforms into a ‘determined’ or ‘committed’ preadipocyte, which then undergoes a secondary differentiation stage to become a lipid laden adipocyte [19, 20]. During the determination stage, multipotent mesenchymal stem cells (MSCs) differentiate and convert to committed preadipocytes under the influence of hormones, insulin, and growth factors [19]. Subsequent stage is mitotic clonal expansion in which growth-arrested preadipocytes undergo several rounds of mitotic division which is a necessary step in the adipocyte differentiation program [21]. Following mitotic clonal expansion, the preadipocytes leave the cell cycle and undergo terminal differentiation, lose their fibroblastic morphology, accumulate cytoplasmic triglyceride, and acquire the metabolic features of mature adipocytes. Adipocyte-specific genes are also highly expressed by mature adipocytes [19]. Adipocyte differentiation is closely regulated by a cascade of transcription factors, amongst which peroxisome proliferator-activated receptor gamma (PPARG) and CAAT/enhancer-binding proteins (C/EBPs) are key players of adipocyte fate.

Furthermore, to achieve successful transformation to mature adipocytes, fibroblastic preadipocytes undergo transformation into spherical cell shape [22, 23]. Proteolytic degradation of the stromal extracellular matrix (ECM) of preadipocyte by the plasminogen cascade is essential for changes in cell morphology, the expression of adipocyte-specific genes, and lipid accumulation [24]. Following changes in ECM, C/EBPα, and PPARG are then activated [25].

### Role of transcription factors in adipogenesis

**Fig. 1:**
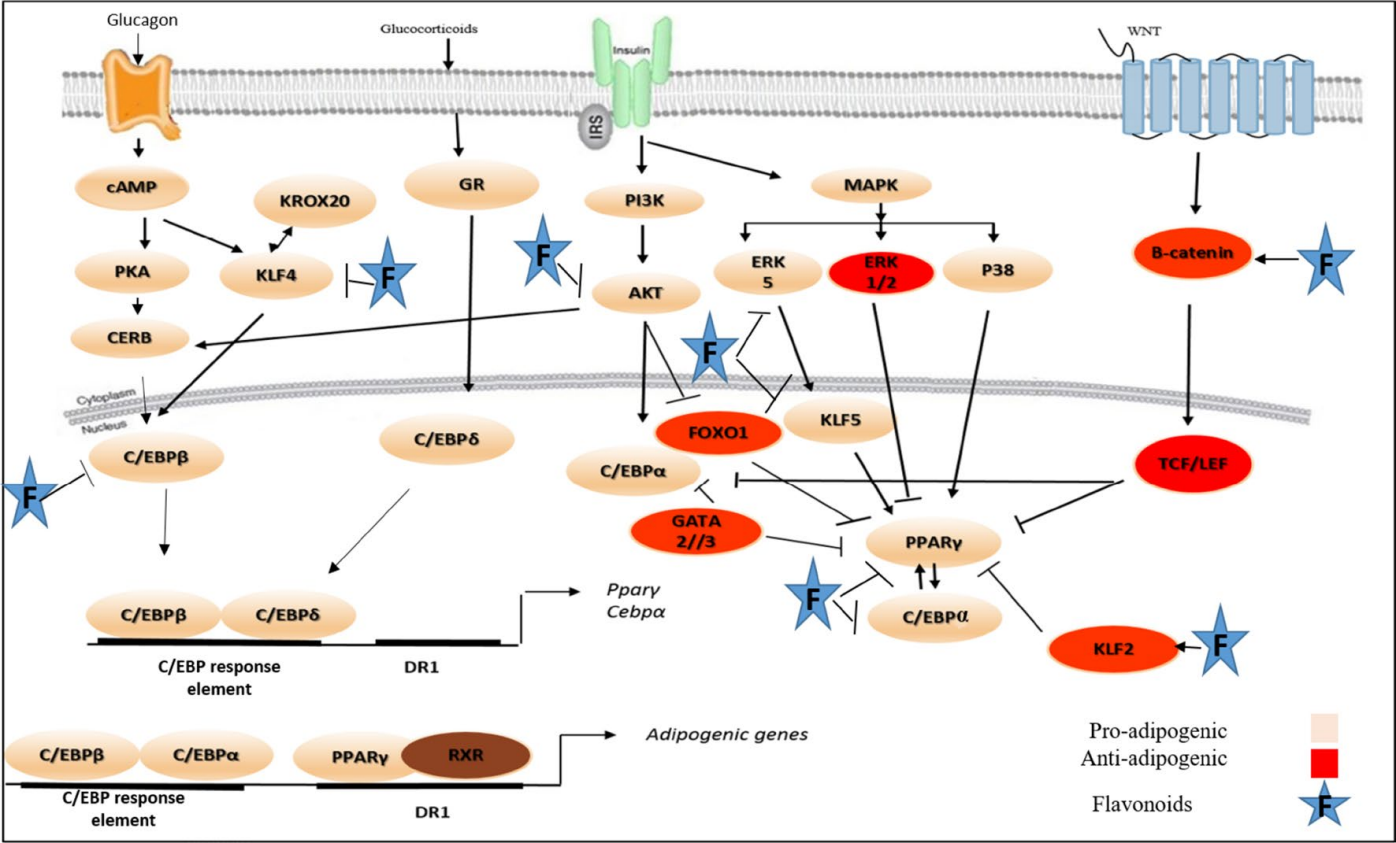
Adipogenesis network. The process of adipogenesis begin with the activation of transcription factors, C/EBPβ and C/EBPδ. These transcription factors function during the early adipogenesis program to regulate the expression of the two master regulators of adipogenesis, PPAR-γ and C/EBPα. The expression of adipogenic genes is regulated by binding of PPARG as a heterodimer with RXRα, where C/EBPα and C/EBPβ occupy the C/EBP response elements. Several other important transcriptional factors play a role in control of adipogenesis. Some transcriptional factors including KLF5 and CREB have a positive role in adipogenesis, whereas other transcriptional factors such as KLF2 and GATA2/3 suppress adipogenesis. *C/EBP* CCAT/enhancer-binding protein, *PPARG* peroxisome proliferator-activated receptor-gamma, *ERK* extracellular signal-regulated kinase, *KLFs* Kruppel-like factors, *CREB* cyclic AMP response element-binding protein, *FOXO1* forkhead box O1, *TCF/LEF* T-cell factor/lymphoid enhancer factor, *MAPK* mitogen-activated protein kinase, *Wnt* wingless-type MMTV integration site family, *PKA* protein kinase A, *GR* glucocorticoid receptor, *DR1* direct repeat type 1 element

Adipogenesis is tightly controlled by the activity of transcription factors which activate or repress each other in a sequential manner. The key transcription factors that are involved in adipogenesis include C/EBP family members (C/EBPα, C/EBPβ, and C/EBPδ) and PPARG. At large, adipogenic program is driven by at least two waves of transcription factors. Adipogenic stimuli (hormones, growth factors, and cytokine) initiated the first wave which amongst others includes C/EBPβ and C/EBPβδ. These proteins subsequently induce expression of the second wave of transcription factors of which PPARG and C/EBPα are the most important (Fig. 1). These two central adipogenic regulators positively control each other and cooperate to orchestrate expression of the full adipogenic program [7].

In addition to the above, an array of other important transcription factors function as regulators of adipogenesis. Krüppel-like factors (KLFs) are expressed in adipose tissue and are either activators or repressors of transcription. KL4, KLF5, KLF6, KLF9, and KLF15 are positive regulators of adipogenesis [26]. KLF5 which is induced early during adipogenesis by C/EBPβ and C/EBPδ activates the *Pparg2* promoter [27]. KLF6 suppresses the expression of preadipocyte factor-1 (Pref-1) which is known to inhibit adipogenesis [28]. Other proadipogenic transcription factors include sterol regulatory element-binding protein 1 (SREBP1) and cyclic AMP response element-binding protein (CREB). SREBP1 promotes early adipocyte differentiation and can induce expression of PPARG and facilitates fatty acid metabolism [29], while the expression of CREB in preadipocytes is necessary to induce adipocyte differentiation. Accordingly, the absence of CREB inhibits adipocytes differentiation [30, 31].

Signals that repress adipocyte development may have profound implications for human health. Amongst the myriad of transcription factors that are known to be repressors of adipocyte differentiation are several members of the KLF (KLF2 and KLF7) [32, 33], globin transcription factor (GATA2 and GATA3), and forkhead (Forkhead Box O1 (FOXO1) and Forkhead Box A2 (FOXA2)) families. GATA2 and GATA3 are known to inhibit terminal differentiation via repressing transcription of PPARG [34].

### Role of transcription cofactors in adipogenesis

Transcription cofactors are proteins that interact with transcription factors and may affect transcription of specific genes in a positive or negative manner and thus play an important role in adipogenesis. Amongst the transcription co-activators, thyroid receptor-associated protein complex 220 (TRAP220) is a known binding partner of PPARG, the absence of which prevents adipocyte differentiation [35]. Other significant co-activators include TATA-binding protein-associated factor-8 (TAF8) which is upregulated during adipogenesis [36]. Hitherto and several other cofactors have been identified to play a role in preadipocyte differentiation that contribute to the intricacies of adipogenesis. The cyclin D3-cyclin-dependent kinase-6 complex can bind to and phosphorylate PPARG, and eventually leads to induction of preadipocyte differentiation [37]. Cyclin-dependent kinase 4 (CDK4) has also been reported to activate PPARG via its kinase domain [38].

By contrast, some cofactors may act as inhibitors of adipogenesis. For instance, cyclin D1 and transcriptional co-activator with PDZ-binding motif (TAZ) suppress PPARG activity and block adipocyte differentiation [39]. Some co-repressors recruit histone deacetylases (HDACs) to target promoters, which in turn result in blockade of transcription. Mammalian sirtuin 1 (SIRT1) with HDAC activity interacts with PPARG and therefore inhibits preadipocyte differentiation. Furthermore, nuclear receptor co-repressors (NCoR) and silencing mediator of retinoid and thyroid hormone receptors can also act as anti-adipogenics [40].

### Cell-cycle proteins

Cyclin-dependent kinases (Cdks) regulate the progression of preadipocytes through the cell division cycle [41]. Cdks when activated phosphorylate retinoblastoma family (Rb) members, including the retinoblastoma protein p130 and p107. This leads to the release of E2 promoter-binding factors (E2Fs) from inhibitory interaction with Rb, enabling E2F family to activate transcription of genes that allows the cells to enter S phase [42]. Cyclins are documented to be downstream targets of c-Myc protein, which has been shown to activate cell cycle and induces DNA synthesis in serum-starved 3T3-L1 cells [43]. Nonetheless, overexpression of c-Myc inhibits differentiation of preadipocytes possibly by inhibiting the cell to enter into a distinct predifferentiation stage in G_0_/G_1_[44]. During conversion from G_1_ to S stage, p38 mitogen-activated protein kinases (MAPKs), extracellular signal-regulated kinase (ERK), and glycogen synthase kinase-3B (GSK3B) phosphorylate and activate C/EBPβ which eventually leads to expression of *Pparg* and *Cebpα* [45].

### Role of microRNAs in adipogenesis

**Fig. 2:**
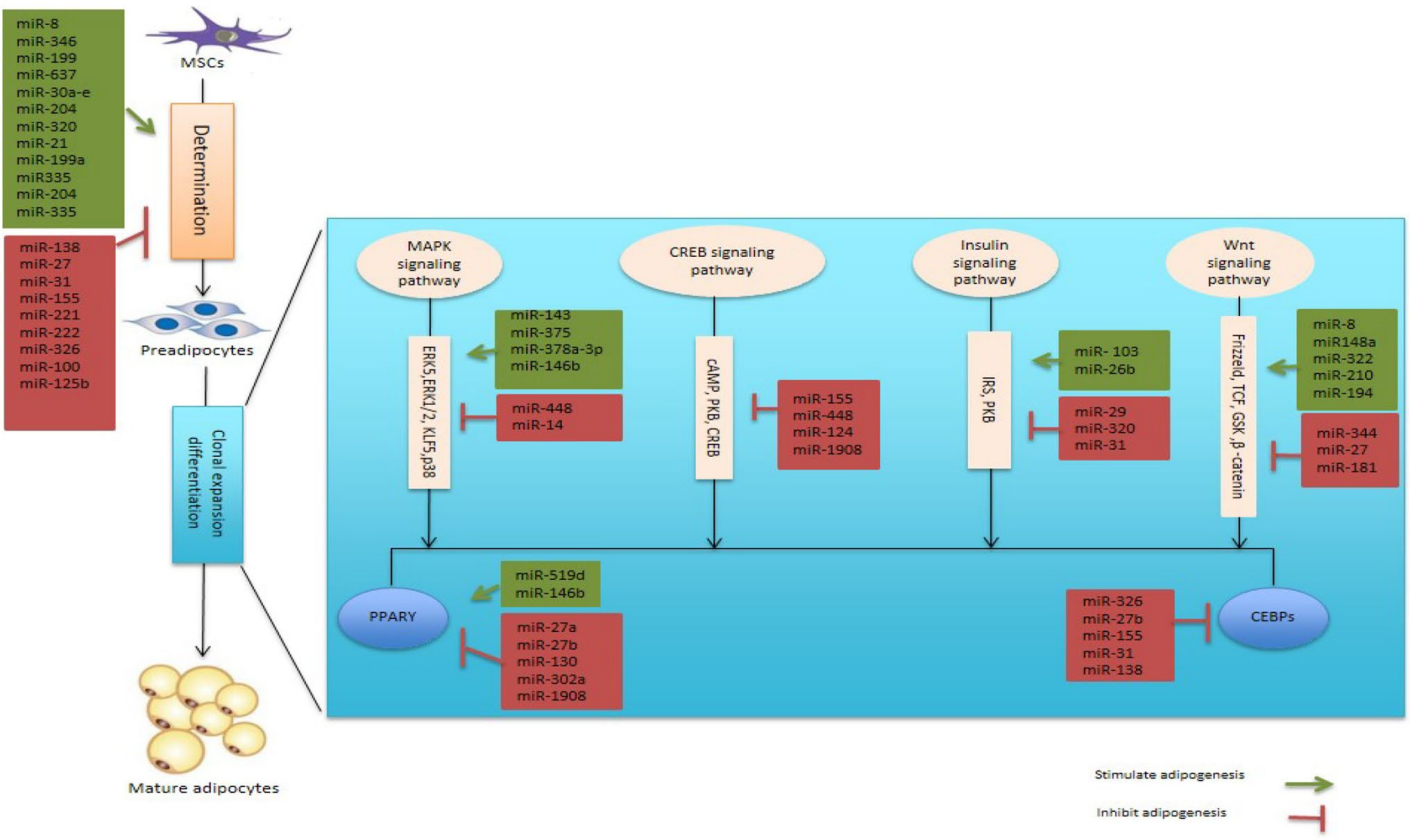
MiRNAs in adipogenesis. MiRNAs influence adipogenesis during determination phase, which is the conversion of mesenchymal stem cell to preadipocytes, clonal expansion, and terminal differentiation of preadipocyte to mature adipocyte. *MAPK* mitogen-activated protein kinase, *ERK* extracellular signal-regulated kinase, *MSC* mesenchymal stem cell, *cAMP* cyclic adenosine monophosphate, *CREB* cAMP response element-binding, *WNT* wingless and INT-1, *TCF* T-cell-specific transcription factor, *PPAR* peroxisome proliferator-activated receptor, *C/EBP* CCAAT/enhancer-binding protein, *KLF* Kruppel-like factor, *IRS* insulin receptor substrate, *PKB* protein kinase B, *GSK* glycogen synthase kinase 3

MicroRNAs (miRNAs) are small non-coding RNAs that regulate different biological processes at post-translational modification state [46]. In addition, they play a role in a variety of human diseases such as obesity and diabetes mellitus [47]. The miRNA profile of human adipose tissue has been demonstrated to be different in obese patients [48–50]. One of the major functions of miRNAs in adipose tissue is to inhibit or stimulate the differentiation of adipocytes. There are numerous inhibitory and promoting miRNAs that contribute to the regulation of adipogenesis, in the commitment stage and in terminal differentiation (Fig. 2). The expression pattern of 70 miRNAs has been shown to be either upregulated or downregulated during adipogenesis in subcutaneous fat cells [49]. In mouse embryonic stem cells, 129 miRNAs expression are altered at distinct time points during conversion of mesodermal progenitor cells to mature adipocyte [51, 52]. MiR-103 is upregulated in rodents epididymal adipocytes during adipogenesis and its ectopic expression increase triglyceride accumulation in the early stage of adipogenesis [53]. However, the expression of miR-103 remains unchanged during adipogenesis in human subcutaneous adipocytes [49]. The reason for the lack of inconsistencies between studies is not known but could be due to differences in fat depots in mice and humans.

MiR-30 family is upregulated during adipogenesis, and it increases adipogenesis via targeting run-related transcription factor 2 (RUNX2) [54]. RUNX2 is an osteogenesis regulator that promotes adipocytes differentiation when it is downregulated. Similarly, miR-204 directly targets RUNX2. Overexpression of miR-204 in MSCs promotes adipogenesis, whereas its inhibition favors osteogenesis [55, 56]. The miR 17–92 cluster is highly expressed during clonal expansion in preadipocytes and enhances adipogenesis through inhibiting the tumor suppressor, Rb2/p130 during the early clonal expansion of preadipocytes [51, 52].

MiRNAs known to inhibit adipogenesis include miR-27, miR-30, microRNA Let-7, and miR-448. Forced expression of miR-27 suppresses adipogenesis in multipotent adipose-derived stem cells and 3T3-L1 cell line [57, 58] by directly targeting *Pparg* and *Cebpα* mRNA [59], whilst miR-130 targets *Pparg*. Others such as microRNA Let-7 inhibit clonal expansion and terminal differentiation in 3T3-L1 cells [60], and miR-448 is reported to inhibit adipogenesis by targeting KLF5 [61].

MiRNAs have also been demonstrated to modulate adipogenesis by targeting Wnt pathway. Wnt proteins are factors in the external environment that can affect the differentiation potential of preadipocytes. MiRNA microarray results revealed increased expression of 18 miRNAs including miR-148a, miR-210, miR-194, and miR-322 that repress Wnt signaling and thus increase adipogenesis [46]. Conversely, 29 miRNAs including miR-27, miR-181, and miR-344 were identified to activate Wnt pathway and suppress adipogenesis [46].

### Role of circadian genes in adipogenesis

It has been documented that in human adipose tissue explants, the circadian genes can oscillate independently of the central nervous system which may regulate the timing of clock-controlled genes such as *Pparg*. Several proteins including nocturnin, period circadian protein homolog 3 (PER3), and brain and muscle Arnt-like protein-1 (BMAL1) that are involved in the regulation of circadian rhythm can influence adipogenesis. Nocturnin, which is a circadian regulated gene, has been reported to facilitate adipogenesis in 3T3-L1 cells via stimulation of PPARG nuclear translocation [62], whereas PER3 was shown to have a negative role in differentiation of MSCs to adipocytes; and besides, the protein can form a complex with PPARG which inhibits PPARG-mediated transcriptional activation via *Pparg* response elements [63]. Similarly, BMAL1 is a negative regulator of adipogenesis. BMAL1 deficiency in mice embryonic fibroblast cells results in increased expression of *Cebpβ* and *Pparg*, and these adipogenic markers are increased even before induction of adipogenesis which suggests spontaneous differentiation of these cells with complete deficiency of BMAL1. BMAL1 has been shown to suppress adipogenesis by direct transcriptional regulation of genes of the Wnt signaling pathway [64]. However, another study showed conflicting results, *Bmal*1 knockout C3H10T1/2 cells failed to be differentiated into mature adipocytes [65].

### Other factors in regulation of adipogenesis

In addition to the role of transcriptional factors, preadipocyte differentiation may be influenced by a number of hormones, growth factors and cytokine. Insulin, insulin-like growth factor-1 (IGF-1), thyroid hormones, mineralocorticoids, glucocorticoids (GCs), and PPARG agonists have important role in promoting adipogenesis. Insulin is an important positive regulator of adipogenesis [66]. Downstream molecules of the insulin signaling cascades such as phosphatidylinositol-3 kinase (PI3K), mammalian target of rapamycin (mTOR), and protein kinase B (PKB) are essential for preadipocyte differentiation [67, 68]. Thyroid hormone (T3), which plays a vital role in the control of metabolic homeostasis, promotes adipogenesis via thyroid receptor α1-induced lipogenic gene expression [69]. Likewise, GCs, which are positive regulator of adipogenesis, promote differentiation of preadipocytes by increasing the expression of *Cebpδ* and *Pparg*. Fibroblast growth factors (FGFs) including FGF1, FGF2, and FGF10 have been shown to have proadipogenic activity, since neutralization of these fibroblast growth factors can block adipogenesis [70–72].

Contrariwise, various extranuclear factors are shown to be negative regulators of adipogenesis. The wingless-type MMTV integration site family (Wnt) acts through autocrine or paracrine manner to regulate cell growth and cell fate in many cell types. Wnt signaling proceeds through canonical (β-catenin) or non-canonical pathways. In the canonical pathway, binding of Wnt to frizzled receptors on the cell surface causes the translocation of β-catenin to the nucleus where it interacts with the T-cell factor/lymphoid enhancer factor (TCF/LEF) transcription factors to inhibit adipogenesis through blockade of C/EBPδ and PPARG [73, 74]. In myometrial tissue, the absence of β-catenin leads to its conversion to adipose tissue, which highlights the importance of Wnt-β-catenin pathway in regulation of adipogenesis [32]. Suggestion has been put forward that the receptors that initiate the Wnt reside on primary cilia on adipocyte surface. This is based on the fact that the increased adipogenesis observed in obese patients with the inherited ciliopathy Bardet–Biedl syndrome may be due to impaired cilia formation which leads to increased expression of PPARG [75]. Transforming growth factor β (TGF-β) is another negative regulator of adipocyte differentiation. TGF-β inhibits adipogenesis through Smad3 which interacts with C/EBPβ and C/EBPδ and represses C/EBP transcription activity [76]. Besides, it may also suppress adipogenesis via induction of c-Myc expression [77]. A soluble form of preadipocyte factor 1 (Pref-1) was shown to reduce adipose tissue mass and this factor negatively regulates adipogenesis via interaction with Notch [78]. Finally, proinflammatory cytokines inhibit adipogenesis via activation of several intracellular signaling pathways. Proinflammatory cytokines were proven to decrease PPARG and C/EBPα expression in preadipocytes and block insulin action [78–80].

Conceivably, PPARG plays a major role in adipogenesis and most of the above factors that influence adipogenesis play their positive or negative role in adipogenesis by ultimately targeting PPARG, and hence, in the next section, we look at PPARG more closely.

### PPARG as a master key of adipogenesis

PPARG which is abundantly expressed in adipose tissue is a master key of adipogenesis [81, 82] and contributes to whole-body insulin sensitivity and glucose homeostasis [81]. Activation of PPARG by ligands such as fatty acids and the antidiabetic drugs, and thiazolidinediones (TZDs) lead to adipocyte differentiation and fatty acid storage. Therefore, intake of high fat food exposes people to prolonged high level of fatty acid (PPARG ligand), which most likely results in obesity [83].

Given that PPARG is an essential regulator of adipogenesis, it has been the target of anti-obesity research. PPARG can be either modulated directly, or indirectly by targeting its upstream factors or pathways which ultimately affect the activity of this crucial regulator of adipogenesis. In this regard, the expression or activity of PPARG can be suppressed through inhibition of C/EBPβ, the increased expression of GATA2 and GATA3, regulation of mitogen-activated protein kinase (MAPK), and the activation of the Wnt/β-catenin pathway. Another group of proteins that may play a regulatory role in PPARG transcriptional activity are the sirtuins (SIRT), especially SIRT1. SIRT1, an NAD^+^-dependent deacetylase, impaired adipogenesis by directly acting as a PPARG co-repressor, thus, counteracting obesity [84, 85]. Still, PPARG may possibly be regulated by post-translational modifications including phosphorylation which, in theory, is a distinct feature that can be subjected for cell- or tissue-specific modulation of this molecule [86, 87]. Phosphorylation of PPARG at Ser273 by CDK5 has been reported to selectively decrease expression of a subset of PPARG-target genes in adipocytes and pharmacological inhibition of Ser273 phosphorylation confers insulin sensitizing effects [88]. Nonetheless, Ser273 phosphorylation does not affect regulation of adipogenesis by PPARG, suggesting that the antidiabetic and proadipogenic roles can be independently manipulated by pharmacological agents.

## Dietary flavonoids

Flavonoids are a class of plant secondary metabolites that are widely distributed in a variety of vegetables and fruits [89]. Flavonoids have a wide range of biological functions, including coloration of flowers, protection against ultraviolet radiation and phytopathogens, signaling during nodulation, and auxin transport [90, 91]. Dietary flavonoids have been shown to possess an array of pharmacological activities including anti-inflammatory, antithrombotic, antitumor, antiviral, anti-atherosclerotic, antidiabetic, and anti-adipogenic effects [92–98].

**Fig. 3:**
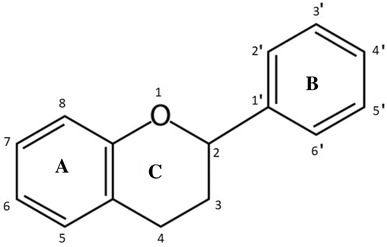
Basic structure of flavonoids

Chemically, flavonoids have the basic structure of a 15-carbon skeleton consisting of two aromatic rings (A and B rings) connected through a heterocyclic pyrane ring (C ring) (Fig. 3). Flavonoids encompass a number of subclasses which are classified based on the level of oxidation and pattern of substitution of the C ring. The six diet-derived flavonoid subclasses include isoflavones, flavan-3-ols, anthocyanidins, flavanones, flavones, and flavonols [99, 100]. Each subclass consist of individual compounds, characterized based on specific hydroxylation and conjugation patterns [99]. The classification of dietary flavonoids, their chemical structures, individual compounds, and dietary source of these subgroups are shown in Table 1.

**Table 1:** Flavonoid subclasses and their dietary sources

Flavonoids	C ring functional group	Dietary source	Compound	Chemical formula
Anthocyanidins	3-Hydroxy	Cherry, berries, and red wine	Cyanidin Delphinidin Malvidin Pelargonidin Petunidin Peonidin	C_15_H_11_O_6_+ C_27_H_31_O_17_+ C_17_H_15_O_7_+ C_15_H_11_O_5_+ C_16_H_13_O_7_+(Cl−) C_16_H_13_O_6_+
Flavones	4-Oxo	Carrots, olive oil, peppers, rosemary peppermint, and celery	Apigenin Luteolin	C_15_H_10_O_5_ C_15_H_10_O_6_
Flavan-3-ols	3-Hydroxy 3-*O*-gallate	Tea, chocolate and cocoa	(+)-Catechin (+)-Gallocatechin (−)-Epicatechin-3-gallate (−)-Epigallocatechin-3-gallate	C_15_H_14_O_6_ C_15_H_14_O_7_ C_22_H_18_O_10_ C_22_H_18_O_11_
Flavonols	3-Hydroxy, 4-Oxo	Onion, olive oil, and berries	Fisetin Isorhamnetin Kaempferol Myricetin Quercetin	C_15_H_10_O_6_ C_16_H_12_O_7_ C_15_H_10_O_6_ C_15_H_10_O_8_
Flavanones	4-Oxo	Citrus fruits	Hesperetin Naringenin	C_16_H_14_O_6_ C_16_H_14_O_5_
Isoflavones	4-Oxo	Soy bean and leguminous plants	Daidzein Genistein Glycitein Biochanin A Formononentin	C_15_H_10_O_4_ C_15_H_10_O_5_ C_16_H_12_O_5_ C_16_H_12_O_5_ C_16_H_12_O_4_

The structure of flavonoids reveals useful information on their anti-adipogenic effect. A comparative study investigated the anti-adipogenic effect of 44 flavonoids in 3T3-L1 cells and concluded that flavonols with a methoxy group at the 3-position possess the strongest anti-adipogenic effect. In addition, the presence of methoxy groups at the B ring contributes to the anti-adipogenic effect of flavonols. On the contrary, flavonoids with hydroxy groups show little or no anti-adipogenic effect [101].

## Interventional studies in adipocyte development by dietary flavonoids

### Anthocyanidins

Anthocyanidins are common plant pigments which are present in many fruits, vegetables, and red wine. To date, about 635 anthocyanin compounds have been identified [102]. Cyanidin, peonidin, malvidin, delphinidin, pelargonidin, and petunidin are the most common anthocyanidins [103]. Human and animal studies have indicated the anti-obesity effect of anthocyanins [92, 103] which have recently attract attention as potential novel anti-adipogenic agents. Cyanidin has been reported to reduce adipogenesis in 3T3-L1 cells by interfering with extracellular matrix and also decreasing carbohydrate response element-binding protein (ChREBP) expression level [93]. Anthocyanins derived from black soybean such as cyanidin-3-*O*-glucoside, delphinidin-3-*O*-glucoside, and petunidin-3-*O*-glucoside have been shown to reduce preadipocyte differentiation through suppression of PPARG [94]. Extracts from *Oryza sativa* L. containing cyanidin-3-*O*-glucoside and peonidin-3-*O*-glucoside have been demonstrated to inhibit the differentiation of mesenchymal C3H10T1/2 cells to preadipocytes [104]. However, a more recent study showed treatment of preadipocytes with black soybean cyanidin-3-glucoside alone paradoxically increases the expression of *Pparg* and *Cebpα*and induces adipogenesis [95]. The discrepancy may be explained by the synergistic anti-adipogenic effects of other anthocyanins present in black soybean extract in the former study [105], where treatment of preadipocytes with the combination of these compounds results in inhibition of adipogenesis. Other possible anti-adipogenic mechanism of anthocyanidins such as cyanidin-3-*O*-glucoside and peonidin-3-*O*-glucoside include activation of Wnt-specific target genes such as *Axin2, Cyclin d1, and Wisp2* [104].

### Flavones

Flavones are present in many herbs including parsley and celery. Apigenin and luteolin are the main dietary flavones [96]. Flavones have shown promising effects in inhibiting adipogenesis. For instance, apigenin suppresses adipogenesis in 3T3-L1 cells via activation of AMP-activated protein kinase (AMPK) pathway [97]. Activation of this pathway has been suggested to inhibit clonal expansion phase and thus adipocyte differentiation [98]. Indeed, apigenin arrests the cell cycle at the G_0_/G_1_ phase which is associated with reduced cyclin D1 and CDK4 expression. Moreover, the exposure of these cells to apigenin reduces expression of PPARG and C/EBPβ [106]. The reduction of C/EBPβ was shown to be due to upregulation of C/EBP inhibitors such as C/EBP homologous protein and the phospho-liver-enriched inhibitory protein isoform of C/EBPβ [107]. Similarly, luteolin inhibits adipogenesis by attenuating the expression of C/EBPα and PPARG. Notably, the PPAR agonist, rosiglitazone-induced adipogenic differentiation in preadipocytes is inhibited by luteolin [108].

### Flavan-3-ols

Flavan-3-ols are widely distributed in human diet and have been shown to be the dominant flavonoid intake by the U.S. adults compared to other flavonoid subclasses [109]. Flavan-3-ols can be found in many fruits including cocoa and tea. The main flavan-3-ols in fruits and cocoa are catechin and epicatechin. Epicatechin gallate (ECG), epigallocatechin (EGC), gallocatechin, and epigallocatechin gallate (EGCG) are mainly present in tea [110]. Many studies demonstrated that catechin possesses anti-adipogenic effect. Tea catechin, in particular (−)-catechin 3-gallate and (−)-epigallocatechin, have been shown to suppress adipogenesis in 3T3-L1 cells via downregulation of PPARG2, C/EBPα, and GLUT4 [111]. However, (−)-catechin derived from green tea has been shown to induce adipocyte differentiation in human bone marrow mesenchymal stem cells (hBM-MSCs) via stimulation of transcriptional activity of PPARG. In addition, the level of adipogenic markers such as adiponectin *(Adipoq*), fatty acid-binding protein 4 (*Fabp4*), and lipoprotein lipase (*Lpl*) are markedly increased. Nevertheless, its stereoisomer (+)-catechin does not show any proadipogenic effect which suggests the possibility of a direct pharmacological target regulated by (−)-catechin [105].

EGCG, the most abundant catechin in green tea, inhibits adipogenesis by reducing the expression of PPARG, C/EBPα, FABP4, and fatty acid synthase while increasing the level of β-catenin in the nucleus. Knocking down of β-catenin using siRNA recovers the expression of these adipogenic markers and attenuates the anti-adipogenic effect of EGCG suggesting Wnt/β-catenin pathway as the anti-adipogenic mechanism of EGCG [112]. EGCG has also been shown to increase apoptosis in mature adipocytes without affecting viability of preadipocytes [113]. However, contradictory result has been obtained with EGCG; Sakuri et al. claimed that EGCG enhances the expression of the genes involved in adipocyte differentiation. The expression of *Pparg1, Pparg2, Cebps*, and *Ppargc1a* was shown to be increased by EGCG treatment. Nonetheless, these effects are only observed at the early and not late stages of adipogenesis [104].

### Flavonols

Flavonols are the most widely distributed flavonoids in the plant kingdom with the exception of algae and fungi. Quercetin, kaempferol, isorhamnetin, fisetin, and myricetin are the main dietary flavonols that can be found in berries, onions, and olive oil. A number of dietary flavonols investigated showed anti-adipogenic effects by interruption of both the conversion of mesenchymal cells to preadipocytes and the differentiation of preadipocytes to mature adipocytes. In vitro studies have indicated that isorhamnetin treatment of preadipocytes results in downregulation of *Pparg* and *Cebpα* without affecting regulation of *Cebpβ* and *Cebpδ*. In addition, this flavonol decreases expression of PPARG-target genes such as liver X receptor-alpha (*Lxr-α*), *Lpl*, and *Fabp4* suggesting PPARG inhibition as a possible mechanism underlying the anti-adipogenic effect of isorhamnetin [114]. Another study conducted by the same group showed that isorhamnetin inhibits differentiation of human adipose tissue-derived mesenchymal stem (hA-MSCs) cells to preadipocytes, wherein it downregulates the mRNA levels of Wnt antagonist such as secreted frizzled-related protein 1 (*Sfrp1*) and dickkopf-1 (*Dkk1*). Furthermore, isorhamnetin stabilizes β-catenin which suggests Wnt signaling pathway as the mechanism responsible for isorhamnetin anti-adipogenic effect in mesenchymal stem cells [115]. Myricetin inhibits hA-MSCs differentiation to preadipocytes and significantly reduces *Pparg*, Cebpα, and *Fabp* gene expression [116]. Besides inhibiting mesenchymal stem cell differentiation, Wang et al. demonstrated the anti-adipogenic effect of myricetin on preadipocyte differentiation. Myricetin-treated 3T3-L1 cells downregulates transcription factors such as *Pparg, Cebpα, Cebpβ, Lpl, Fabp4, Glut4, and Srebp-1c*. Other anti-adipogenic targets of myricetin include inhibition of ERK and c-Jun N-terminal kinase (JNK) phosphorylation during the differentiation process [117]. Microarray analysis revealed that kaemperol decreases expression of adipogenic transcription factors and triglyceride synthesis-related genes and, conversely, increases gene involved in lipolysis [106]. Many transcriptional factors such as C/EBPβ, KLF4, and KLF5 are downregulated by kaempferol treatment, while negative regulators of adipogenesis such as KLF2 and Pref-1 are upregulated during the early adipogenesis [118]. As with many flavonoids, the anti-adipogenic mechanisms of kaempferol are several. Kaempferol also prevents adipocyte differentiation by inhibiting cell-cycle progression via regulation of cyclins. In addition, kaempferol treatment during early adipogenesis inhibits phosphorylation of AKT and mTOR signaling pathways. Fisetin was reported to induce cell-cycle arrest in preadipocytes by suppressing cell-cycle regulatory proteins such as cyclin A, cyclin D1, and CDK4 expression [119]. Nonetheless, another study reported that fisetin reduces adipogenesis by suppression of mTORC1 activity, wherein the flavonol inhibits mTOR phosphorylation and its downstream molecules such as p70 ribosomal S6 kinase which in turn decreases Cebpα gene expression [120, 121]. Rhamnetin blocks adipocyte differentiation during the early stage of adipogenesis program by inhibition of clonal expansion. The expression level of adipogenic transcription factors in the presence of rhamnetin also declines during the early adipogenesis [122].

A comparative study investigated and compared the inhibitory effects of flavonoids (rutin, naringenin, hesperidin, quercetin, naringin, and resveratrol) on adipocyte differentiation, as indicated by the decreases in triglyceride accumulation and GPDH activity [123]. In this study, rutin, a flavonol glycoside, exhibits the highest anti-adipogenic effect characterized by downregulation of adipogenic transcription factors and leptin, and upregulation of adiponectin [124]. Choi et al. investigated the anti-adipogenic effect of rutin in preadipocytes and HFD-induced obese animals. The results indicates that rutin decreases the expression of key adipogenic transcription factors. Experimental animals which received rutin gain less body weight and have lower blood cholesterol [125]. However, rutin was shown to be slowly and poorly absorbed in human [126] as it has to be hydrolysed by the intestinal microflora. These findings may limit the effectiveness of rutin as anti-adipogenic agent following dietary consumption.

Several mechanisms contribute to the anti-adipogenic effect of quercetin. Exposure of mouse preadipocytes to quercetin leads to activation of AMPKα and β1 and phosphorylation of their substrate, acetyl-CoA carboxylase. In addition, quercetin reduces adipocyte differentiation by inhibiting clonal expansion during the early adipogenesis via suppression of cyclin A [127]. A recent study indicated that quercetin prevents differentiation of OP9 mouse stromal cells into mature adipocytes through downregulation of adipogenic transcription factors, FABP4, LPL, and upregulation of adipose triglyceride and hormone sensitive lipases [128]. Quercetin may reduce adipose tissue mass not only by inhibiting adipogenesis, but this flavonol also induces apoptosis in mature adipocytes by modulating mitogen-activated protein (MAP) kinases, in particular ERKs and JNK pathway [129].

### Flavanones

Flavanones are a subclass of dietary flavonoids that are found to be rich in citrus fruits. The major dietary flavanones are naringin and hesperidin [110]. Although naringin and hesperidin have shown promising effects in preventing obesity, limited studies have been conducted to investigate the anti-adipogenic effect of these flavanones.

Hesperetin, an aglycone of hesperidin, inhibits adipogenesis in hBM-MSCs by reducing resistin (*Retn*), *Adipoq, Fabp4, Pparg, Stat5a, Lpl*, and tumor necrosis factor-alpha (*Tnf-α*) expression, while increasing proapoptotic genes such as *Bcl* and *Bax* in preadipocytes. This study suggests inhibition of adipocyte differentiation in hBM-MSCs and induction of apoptosis in preadipocytes responsible for the anti-adipogenic effect of hesperetin [130].

### Isoflavones

Dietary isoflavones are present in legumes, soy bean, and soy foods. As with many natural products, isoflavones such as genistein and daidzein can target more than one pathways in the development of adipocytes. Cultured human adipose-derived mesenchymal cells (hAD-MSCs) treated with genistein or daidzein maintain their fibroblast-like appearance and express Oct-4, the stem cell marker indicating the differentiation of these cells into preadipocytes is interrupted. Once the cells become committed to adipose lineage, genistein demonstrates anti-adipogenic effect by inhibition of *Pparg, Srebp-1c*, and *Glut 4* during intermediate phase of the adipogenesis program. Microarray result indicated that activation of Wnt pathway through estrogen receptor (ER)-dependent pathways including ERK/JNK signaling and LEF/TCF4 co-activators are amongst the mechanisms underlying the anti-adipogenic effect of genistein [131]. Recent studies have shown that hypoxic suppressions of adipocyte differentiation are associated with AMPK activation which, in turn, can impair mitotic clonal expansion during the early adipogenesis. In this context, genistein has been demonstrated to induce reactive oxygen species (ROS), which eventually leads to activation of AMPK and inhibition of mitotic clonal expansion. Furthermore, genistein was shown to activate AMPK comparable to 5-amino-imidazole-4-carboxamide riboside (AICAR), a known activator of AMPK. Both genistein and daidzein also stimulate lipolysis [131]. Another study investigated the underlying mechanisms responsible for the anti-adipogenic effect of genistein. Genestein has been demonstrated to stimulate lipolysis by preventing the inhibitory effect of dexamethasone on eNOS expression and NO release in 3T3-L1 cells. In addition, pretreatment of preadipocytes with genistein has been reported to inhibit fatty acid synthase and suppress p38; expression of fatty acid synthase is associated with activation of p38 [119]. Indeed, phosphorylation of p38 mitogen-activated protein kinase is required for adipocyte differentiation during the early adipogenesis, wherein treatment of preadipocytes with p38 inhibitors suppressed adipogenesis [120]. Genistein also affects other pathway during adipocyte development; this flavonoid inhibits janus-activated kinase (JAK) 2 to attenuate the effect of growth hormones in promoting adipogenesis [121]. In addition, genistein was shown to suppress adipogenesis by induction of apoptosis in mature adipocytes [132, 133].

Recently, SIRTs, specifically SIRT1, have become a focus of intense anti-obesity research [134]. The NAD+-dependent deacetylase SIRT1 has been shown to maintain proper metabolic functions in many tissues to protect against obesity [84]. As a matter of fact, SIRT1 inhibits adipogenesis by repressing the transcriptional activity of PPARG [85]. Furthermore, mice with adipose tissue-specific SIRT1 deletion exhibit greater adiposity and metabolic dysregulation, including insulin resistance [135]. Other study investigated the role of SIRT1 in curbing adipocyte hyperplasia; SIRT1 knockdown results in hyperplastic, small, and inflamed adipocytes that appear to be dysfunctional metabolically and physiologically [136]. This study demonstrated that reduced levels of SIRT1 cause c-Myc to become hyperacetylated, which leads to higher preadipocyte proliferation potential and enhanced adipocyte mitotic clonal expansion during differentiation, which eventually results in dysfunctional hyperplastic adipocytes. Indeed, SIRT1 levels are reduced in mice-fed high fat diet which triggers inflammation-induced cleavage and inactivation of SIRT1 [135]. In this context, some of the anti-adipogenic effects of flavonoids mentioned above may very well be due to their actions on SIRT1; quercetin and apigenin have been shown to increase NAD + levels which leads to activation of SIRT1 [137]. It has been reported that resveratrol inhibits human preadipocyte proliferation and adipogenesis in a SIRT1-dependent manner [116]. Fisetin suppresses the early stages of adipogenesis through SIRT1-mediated deacetylation of PPARγ and FoxO1, and enhances the association of SIRT1 with the PPARγ promoter, leading to suppression of PPARγ transcriptional activity [117].

Collectively, flavonoids exert their beneficial effects against adipogenesis through multiple pathways (Table 2). Although these findings are encouraging, most of their anti-adipogenic effects are identified from cellular models of adipogenesis and remains to be validated in vivo or in human cells. We must also keep in mind that much of these data are based on rodent models which cannot always be directly extrapolated to clinical effects. However, such studies elucidate different molecular mechanisms by which flavonoids, either as individual treatments or in combination, might be effective in prevention of adipocyte differentiation and ultimately obesity.

**Table 2:** List of flavonoids and their underlying mechanisms of action in adipogenesis

Flavonoids	Stages of adipogenesis	Effect	Pathways/target molecules	Experimental model and dose applied	Comments	References
Anthocyanidins						
Cyanidin	Terminal differentiation	Anti-adipogenic	Upregulation of ChREBP and interfering with the extracellular matrix	Preadipocytes obtained from subcutaneous and visceral human adipose explant tissue	Reduces adipogenesis via interfering with extracellular matrix and decreasing ChREBP expression level	[93]
Cyanidin-3-*O*-glucoside	Terminal differentiation	Proadipogenic	Upregulation of *Pparg* and *Cebpα*	3T3-L1 cells (20 and 100 µM)/*db*/*db* mice (black soy bean extract 30 mg/kg, orally)	Increases *Pparg* and *Cebpα* expression, adiponectin secretion and activates insulin signaling cascade	[95]
	Determination	Anti-adipogenic	Activation of Wnt pathway	C3H10T1/2 cells (black rice extract 10, 20, 40, and 80 µg/ml)/HFD mice (black rice extract, 100 mg/kg, orally)	Inhibits differentiation of mesenchymal cells to preadipocytes and induces Wnt-specific target genes such as *Axin2, Wisp2*, and *Cyclin d1*	[138]
	Terminal differentiation	Anti-adipogenic	Suppression of PPARG	3T3-L1 cells (black soybean extract 12.5 and 50 µg/ml)	Reduces lipid accumulation and suppresses PPARG expression	[94]
Delphinidin-3-*O*-glucoside	Terminal differentiation	Anti-adipogenic	Suppression of PPARG	3T3-L1 cells (black soybean extract 12.5 and 50 µg/ml)	Reduces lipid accumulation and suppresses PPARG expression	[94]
Peonidin-3-*O*-glucoside	Determination	Anti-adipogenic	Activation of Wnt pathway	C3H10T1/2 cells (black rice extract 10, 20, 40, and 80 µg/ml)/HFD mice (black rice extract, 100 mg/kg, orally)	Inhibits differentiation of mesenchymal cells to preadipocytes and induces Wnt-specific target genes such as *Axin2, Wisp2*, and Cyclin D1	[138]
Flavones						
Apigenin	Clonal expansion	Anti-adipogenic	Inhibition of mitotic clonal expansion and cell-cycle arrest	3T3-L1 cells (30 and 70 µM)	Inhibits clonal expansion, arrests cell cycle at the G_0_/G_1_ phase and decreases PPARG and C/EBPβ levels	[107]
	Terminal differentiation	Anti-adipogenic	Activation of AMPK	3T3-L1 cells (10, 50 µM)	Induces activation of AMPK and decreases expression of adipogenic and lipolytic genes	[97]
Luteolin	Terminal differentiation	Anti-adipogenic	Inhibition of the transactivation of PPARG	3T3-L1 cells	Attenuates PPARG and C/EBPα expression	[108]
Baicalein	Clonal expansion	Anti-adipogenic	Suppression of Akt-C/EBPα-GLUT4 signaling	3T3-L1 cells (50 µM)	Decreases the intracellular lipid accumulation by downregulation of glucose uptake via repression of Akt-C/EBPα-GLUT4 signaling	[133]
Flavan-3-ols						
Catechin	Terminal differentiation	Anti-adipogenic	Suppression of PPARG2, C/EBPα& GLUT4	3T3-L1 cells (50, 75, 100 µM) 3T3-L1 cells (30 µM)	Inhibits adipogenesis via suppression of PPARG2, C/EBPα, and GLUT4	[111, 139]
(−)-Catechin	Determination	Proadipogenic	Upregulation of *Pparg*	hBM-MSCs (1 and 100 µM)	Upregulates the mRNA levels of adipogenic markers, such as *Adipoq, Pparg, Fabp4*, and *Lpl* in hBM-MSCs	[105]
(−)-Epigallocatechin gallate	Terminal differentiation	Proadipogenic	Upregulation of *Pparg* and *Cebps*	3T3-L1 cells (0.5, 5, or 10 µM)	Increases expression of *Pparg1, Pparg2*, and *Cebps*	[104]
(−)Epigallocatechin gallate	Terminal differentiation	Anti-adipogenic	Activation of Wnt/β-catenin pathway	3T3-L1 cells (100, 150, 200 µM)	Reduces expression of adipogenic markers such as PPARG, C/EBPα, FABP4 and fatty acid synthase while increases β-catenin in the nucleus	[112]
	Terminal differentiation	Anti-adipogenic	Apoptosis	3T3-L1 cells (50–200 µM)	Increases apoptosis in mature adipocytes without affecting viability of preadipocytes	[113]
Flavonols						
Fisetin	Terminal differentiation	Anti-adipogenic	Inhibition of mTORC1 signaling	3T3-L1 cells (50 µM) /HFD mice (HFD supplemented with 0.2% or 0.5% (w/w) fisetin)	Reduces adipogenesis by suppression of mTORC1 activity	[140]
	Clonal expansion	Anti-adipogenic	Inhibition of mitotic clonal expansion	3T3-L1 cells (10, 30 µM)	Suppresses cell cycle regulatory proteins such as cyclin A, cyclin D1 and CDK4 expression and inhibits cell proliferation	[141]
	Terminal differentiation	Anti-adipogenic	Inhibition of mTOR-C/EBPα Signaling	3T3-L1 cells (10 µM)	Downregulates *Pparg* and *Cebpα*expression during adipogenesis	[142]
Isorhamnetin	Terminal differentiation	Anti-adipogenic	Suppression of *Pparg*	3T3-L1 cells (50 µM)	Reduces *Pparg* and *Cebpα* expression. However *Cebpβ* and *Cebpδ* expression remains unchanged	[114]
	Determination/terminal differentiation	Anti-adipogenic	Stabilization of β-catenin protein	hA-MSCs/3T3-L1 cells (1, 25 µM)	Inhibits Wnt receptor and co-receptor genes expression, increases β-catenin, but no effect on *β-catenin* mRNA levels	[115]
Kaempferol	Clonal expansion	Anti-adipogenic	Inhibition of cell-cycle progression, AKT and mTOR signaling pathway	3T3-L1 cells (30 µM)/zebra fish (5, 10, 20 µM)	Inhibits cell-cycle progression via regulation of cyclins. In addition, inhibits phosphorylation of AKT and mTOR signaling pathway. Many transcriptional factors such as C/EBPβ, KLF4 and KLF5 are downregulated, while nKLF2 and Pref-1 are upregulated	[118]
	Terminal differentiation	Anti-adipogenic	Suppression of *Pparg*	3T3-L1 cells (40, 80 µM)	Downregulates expression of *Pparg, Cebpβ, Srebp1, Rxrβ, Lxrβ, Rorα* and also genes involved in triglyceride biosynthesis such as *Gpd1, Agpat2, Dgat2*	[106, 143]
Myricetin	Terminal differentiation	Anti-adipogenic	Suppression of *Pparg*	3T3-L1 cells (100 µM)	Reduces expression of Cebpα, *Pparg*, Cebpβ, *Sreb1c, Fabp4, Lpl* and *Glut4*. Also, inhibits phosphorylation of ERK and JNK during differentiation of adipocytes	[144]
	Determination	Anti-adipogenic	Suppression of *Pparg*	hA-MSCs (30 µM)	Reduces expression of *Cebpα, Pparg, Lpl* and *Fabp*	[145]
Rhamnetin	Clonal expansion/terminal differentiation	Anti-adipogenic	Inhibition of mitotic clonal expansion, and suppression of *Pparg*	3T3-L1 cells	Decreases expression of *Pparg,Cebpα*, and perilipin (*Plin*) Also completely inhibits triglyceride biosynthesis and clonal expansion	[122]
Rutin	Terminal differentiation	Anti-adipogenic	Suppression of *Pparg* and *Cebpα*	3T3-L1 cells (0.25, 0.5, 1.0 mg/ml)/3T3-L1 cells (10, 30, 100 µM)/HFD mice (25, 50 mg/kg, orally)	Reduces mRNA expression of *Pparg* and *Cebpα*	[125, 146]
Quercetin	Terminal differentiation	Anti-adipogenic	Activation of AMPK pathway	3T3-L1 cells (10, 50, 100 µM)	Stimulates activation of AMPK pathway and phosphorylation of acetyl-CoA carboxylase	[129]
	Clonal expantion	Anti-adipogenic	Inhibition of mitotic clonal expansion	3T3-L1 cells (50, 100 µM)	Decreases adipocyte differentiation by inhibition of clonal expansion during early adipogenesis via suppression of cyclin A	[147]
	Determination	Anti-adipogenic	Suppression of PPARG, C/EBPα and SREBP-1c	OP9 mouse stromal cells (50 µM)	Downregulates mRNA and protein expression of C/EBPα, PPARG, SREBP-1c, FAS, FABP4 and mRNA level of *Hsl* and *Lpl*	[148]
Flavanones						
Hesperetin	Determination	Anti-adipogenic	Apoptosis	hBM-MSCs (10, 20, 40, 80, 160 µM)	Decreases expression of *Adipoq, Retn, Fabp4, Pparg, Lpl* and *Tnf-α*, while upregulates proapoptotic genes	[130]
Naringenin	Terminal differentiation	Anti-adipogenic	Suppression of PPARG	3T3-L1 cells (25, 50 µg/ml)	Reduces expression of FABP4, PPARG, STAT5A, and adiponectin	[149]
Isoflavones						
Biochanin A	Determination	Anti-adipogenic	Suppression of *Pparg*	hA-MSCs (0.1, 0.3, and 1 µM)	Decreases *Pparg*, Leptin *(Lep)*, osteopontin (*Opn*)and *Lpl* expression	[150]
Daidzein	Determination	Anti-adipogenic	Stimulation of lipolysis	hA-MSCs (0.1–100 µM)	Stimulates lipolysis by cAMP-dependent protein kinase-mediated hormone sensitive lipase	[131]
Formononetin	Terminal differentiation	Proadipogenic	Upregulation of PPARG	C3H10T1/2 cells (1–20 µM)	Upregulates PPARG and its target genes such as *Fabp4* and *Glut4*	[151]
	Terminal differentiation	Proadipogenic	Upregulation of PPARG	3T3-L1 cells (1–20 µM)	Upregulates PPARG and its target genes such as *Fabp4* and *Glut4*	[152]
Genistein	Clonal expansion	Anti-adipogenic	Inhibition of mitotic clonal expansion and PPARG expression	3T3-L1 cells (5, 50, 100 µM)	Blocks adipogenesis by inhibition of mitotic clonal expansion and suppression of PPARG expression	[153]
	Terminal differentiation	Anti-adipogenic	Suppression of MAPK	3T3-L1 cells (50 µM)	Increases eNOS expression, inhibits phosphorylation of JAK2 and decreases FAS expression	[119]
	Clonal expansion/terminal differentiation	Anti-adipogenic	Activation of AMPK and induction of apoptosis and inhibition of clonal expansion	3T3-L1 cells (100 µM)	Induces ROS, activates AMPK and inhibits mitotic clonal expansion	[154]
	Determination	Anti-adipogenic	Activation of Wnt signaling via ERs-dependent pathway	hA-MSCs (0.1–100 µM)	Activates Wnt/β-catenin pathway, inhibits expression of *Pparg, Srebp-1c* and *Glut4*	[131]

## Final remarks and conclusion

Prolonged excessive energy intake without an increase in energy expenditure promotes the increase in adipocyte size and number. Hyperplasia is triggered by a network of signaling factors that induce conversion of MSCs to preadipocytes which then differentiate into mature adipocytes. Interrupting adipogenesis at any stage of adipocyte differentiation may serve as potential therapeutic strategy against adipogenesis and obesity. In this context, dietary flavonoids have become the subject of increasing scientific interest due to their effects on adipogenesis. The anti-adipogenic effects of flavonoids are mainly via their effect on a number of molecular targets and regulation of several pathways such as induction of apoptosis [123], suppression of key adipogenic transcription factors [94, 95, 114, 144], activation of AMPK [97, 129], and Wnt [115, 138] pathways, inhibition of clonal expansion [147, 153, 155], cell-cycle arrest, and modulation of insulin signaling cascade [118], suggesting flavonoids as effective inhibitors of adipogenesis during determination and terminal differentiation stages.

Although these data are encouraging, further investigation is essential to gain insight into the molecular mechanisms that connect extranuclear and nuclear mediators of adipogenesis. Even though most of the studies have shown that flavonoids suppress the key adipogenic regulators such as PPARG and C/EBPα, the upstream mechanisms which led to suppression of these master regulators of adipogenesis are not fully investigated. In addition, the effect of flavonoids in areas such as microRNAs and circadian clock need to be more explored.

Strategies to develop flavonoids as treatment or as supplementary treatment of obesity will have to consider the pharmacokinetic aspects of the molecules, as well. We have to bear in mind that the in vitro evidence of flavonoids in suppressing adipogenesis might be somewhat of limited impact due to the fact that in vivo flavonoids are extensively metabolized to molecules with different structures and activities and, therefore, may preclude their use in humans [127]. Flavonoids are substrates for conjugating and hydrolyzing enzymes in the small intestine, liver, and colon. Conjugation of flavonoids first occurs in the small intestine followed by the liver where they are further metabolized and the produced glucuronides and sulfate derivatives facilitate their excretion via urine and bile. The compounds that are not absorbed in the intestine will reach the colon and be subjected to structural modifications by colonic microflora. The flavonoid glucuronides that re-enter the enterohepatic circulation through bile excretion are hydrolyzed by the gut microbiota to aglycones that can further be catabolized to low-molecular-weight compounds that can readily be absorbed [128]. This bacterial conversion of flavonoids may have potential health consequences for the host. Therefore, the differences in intestinal microbiota composition among different species may result in different profiles of flavonoid metabolites with different bioactivity [156]. This emphasizes the importance of studying the pharmacokinetic profile of the various flavonoids in human subjects.

It is also important to determine the amount of flavonoids or bioactive compounds in foods or dietary supplement as well as their bioavailability. Despite the health benefits of flavonoids, the bioavailability of flavonoids is generally low and can vary drastically among different flavonoid classes as well as among compounds in a particular class. Flavonoids with complex structures and larger molecular weights may even have lower bioavailability [128]. In human diet, the concentration of flavonoids may be too low to generate adequate efficacy for their health benefits including anti-adipogenic properties. During the past few decades, dietary supplements have become increasingly popular as an alternative source to flavonoid-rich fruits and vegetables [157]. Even though consuming supplements can ensure us that we are getting our daily dose of flavonoids, toxicity issues as well as nutrient–drug interactions should be the subject of concern. Furthermore, the health promoting effects of some of dietary flavonoids are due to the synergistic effects of other flavonoids or compounds present in the food. Therefore, this complex mixture of secondary plant metabolites cannot be simply replaced by purified molecules as dietary supplements. Further investigations on the synergistic effects of flavonoids on adipogenesis are required. There is also a need for more studies assessing flavonoid absorption, organ- or tissue-specific distribution, and accumulation. Specifically, the availability of flavonoids or active metabolites to adipose tissues depends, amongst others, on the lipid solubility of the substance. Flavonoids possessing a number of unsubstituted hydroxyl group and glycosides are polar and water-soluble. There is a negative correlation between the number of hydroxyl groups and the lipophilicity of flavonoids. Although most flavonoids are water-soluble, they possess some lipophilicity, as well. It is well known that flavonoid aglycones are only slightly soluble in water and show lipophilic properties. This lipophilic behavior of flavonoid aglycones may allow the uptake of flavonoids by adipose tissue. For instant, quercetin and its metabolites (isorhamnetin and tamarixetin) in their aglycone form were found in variable amount in most tissues in rat including white adipose tissue [132]. However, the lowest concentration was found in adipose tissue and brain [132]. Nevertheless, long-term intake of flavonoid-rich diet or supplements may result in adequate absorption and accumulation of anti-adipogenic concentration of flavonoids in adipose tissues.

Even though flavonoids have shown promising effect on inhibiting adipogenesis under experimental conditions, low bioavailability of some flavonoids needs to be enhanced for full exploitation of their benefits in prevention of adipogenesis. Therefore, more investigations on the appropriate dose of flavonoid in supplements and also methods to improve bioavailability and thus efficacy of certain flavonoids are warranted.

## Abbreviations

FABP4: Fatty acid-binding protein 4
hBM-MSCs: Human bone marrow mesenchymal stem cells
LPL: Lipoprotein lipase
ChREBP: Carbohydrate response element-binding protein
EGCG: Epigallocatechin gallate
FASN: Fatty acid synthase
GPDH: Glycerol-3-phosphate dehydrogenase
T2DM: Type 2 diabetes mellitus
C/EBP: CCAT/enhancer-binding protein
Cdks: Cyclin-dependent kinases
Rb: Retinoblastoma
ERK: Extracellular signal-regulated kinase
KLFs: Kruppel-like factors
Pref-1: Preadipocyte factor-1
CREB: Cyclic AMP response element-binding protein
EPAS1: Endothelial PAS domain protein 1
BMAL1: Brain and muscle Arnt-like protein 1
FOXO1: Forkhead box O1
FOXA2: Forkhead box A2
TRAP220: Thyroid receptor-associated protein complex 220
TAF8: TATA-binding protein-associated factor-8
HDACs: Histone deacetylases
NCoR: Nuclear receptor co-repressors
mTOR: Mammalian target of rapamycin
ADD-1: Adipocyte determination and differentiation-dependent factor 1
TCF/LEF: T-cell factor/lymphoid enhancer factor
TGF-β: Transforming growth factorβ
ECG: Epicatechin gallate
EGC: Epigallocatechin
LXR-alpha: Liver X receptor-alpha
hA-MSCs: Human adipose tissue-derived mesenchymal stem
JNK: C-Jun N-terminal kinase
HFD: High fat diet
MAP: Mitogen-activated protein
AICAR: 5-Amino-imidazole-4-carboxamide riboside
JAK: Janus-activated kinase
miRNA: MicroRNA
Wnt: Wingless-type MMTV integration site family
AMPK: AMP-activated protein kinase
SFRP1: Secreted frizzled-related protein 1
Dkk1: Dickkopf-1
PPARG: Peroxisome proliferator-activated receptor-gamma
SREBP1: Sterol regulatory element-binding protein 1
FGFs: Fibroblast growth factors
RUNX2: Run-related transcription factor 2
PKB: Protein kinase B
TNF-α: Tumor necrosis factor-alpha
